# RETRACTION: Silencing CDR1as Enhances the Sensitivity of Breast Cancer Cells to Drug Resistance by Acting as a miR‐7 Sponge to Down‐Regulate REGγ

**DOI:** 10.1111/jcmm.71247

**Published:** 2026-06-23

## Abstract

**RETRACTION**: YangW. , X. YangX., Wang, GuJ. , ZhouD. , WangY. , YinB. , GuoJ. and ZhouM. , “Silencing CDR1as Enhances the Sensitivity of Breast Cancer Cells to Drug Resistance by Acting as a miR‐7 Sponge to Down‐Regulate REGγ,” Journal of Cellular and Molecular Medicine 23, no. 8 (2019): 4921‐4932, 10.1111/jcmm.14305.31245927 PMC6652952

The above article, published online on 27 June 2019 in Wiley Online Library (wileyonlinelibrary.com), has been retracted by agreement between the journal Editor‐in‐Chief, Stefan N. Constantinescu; the Foundation for Cellular and Molecular Medicine; and John Wiley & Sons Ltd. The retraction has been agreed following concerns raised by a third party. An investigation identified multiple instances of duplication within Figures 2B, 5B and 6B as well as duplication between Figures 2B and 5B. The authors were contacted and invited to comment on the concerns and to provide supporting data, but no response was received. The editors no longer have confidence in the reliability of the findings reported in the article. The authors did not respond to our notice of retraction.

**RETRACTION**: W. Yang , X. Yang, X. Wang, J. Gu , D. Zhou , Y. Wang , B. Yin , J. Guo and M. Zhou , “Silencing CDR1as Enhances the Sensitivity of Breast Cancer Cells to Drug Resistance by Acting as a miR‐7 Sponge to Down‐Regulate REGγ,” Journal of Cellular and Molecular Medicine 23, no. 8 (2019): 4921‐4932, 10.1111/jcmm.14305.31245927 PMC6652952

The above article, published online on 27 June 2019 in Wiley Online Library (wileyonlinelibrary.com), has been retracted by agreement between the journal Editor‐in‐Chief, Stefan N. Constantinescu; the Foundation for Cellular and Molecular Medicine; and John Wiley & Sons Ltd. The retraction has been agreed following concerns raised by a third party. An investigation identified multiple instances of duplication within Figures 2B, 5B and 6B as well as duplication between Figures 2B and 5B. The authors were contacted and invited to comment on the concerns and to provide supporting data, but no response was received. The editors no longer have confidence in the reliability of the findings reported in the article. The authors did not respond to our notice of retraction.

